# How to keep your cool: heat tolerance and thermoregulatory strategies of a cold adapted insectivorous bat

**DOI:** 10.1007/s00442-025-05776-3

**Published:** 2025-07-28

**Authors:** Ruvinda K. de Mel, Sanjeev Baniya, Zenon J. Czenze

**Affiliations:** Centre for Behavioural and Physiological Ecology, University of New England, Armidale, NSW 2351 Australia

**Keywords:** Thermoregulation, Evaporative water loss, Heat tolerance, Small endotherms, Bats, Climate change

## Abstract

Global climate change exposes small endotherms to high ambient temperatures (*T*_a_), increasing the risk of dehydration and hyperthermia. These threats are disproportionately severe on cold adapted small endotherms with limited heat tolerance. Using temperature telemetry and flow through respirometry, we investigated the thermoregulatory strategies of lesser long-eared bats (*Nyctophilus geoffroyi*) inhabiting a temperate ecosystem. We predicted this population would have low heat tolerance limits and would initiate active thermoregulation at low *T*_a_s. We also predicted that, similar to other populations, females would have higher heat tolerance limits and a more conservative water economy than males given their reproductive ecology places a high demand on water reserves. In line with our predictions, the subcutaneous temperature (*T*_sub_), whole animal resting metabolic rate (waRMR) and whole animal evaporative water loss (waEWL) data suggest that they experience heat stress at low *T*_a_s. Females could tolerate higher experimental *T*_a_s (females = 44 ℃, males = 42 ℃) and initiated evaporative cooling at a significantly higher *T*_a_ (37.7 ℃) than males (33.1 ℃), which is considerably lower than other populations of the same species adapted to arid environments. Our results highlight how different environment conditions and contrasting reproductive ecologies between sexes influence the thermoregulatory capacities and strategies of small endotherms. Although this population does not currently face consistent threats from lethal hyperthermia or dehydration, increasing *T*_a_s could expose them to sub-lethal fitness costs such as progressive mass loss due to higher energy expenditure for thermoregulation.

**Supplementary Information:**

The online version contains supplementary material available at 10.1007/s00442-025-05776-3.

## Introduction

Climate change induced increases in air temperatures (*T*_a_) will increase thermoregulation costs at high *T*_a_s for small endotherms, with many predicted to experience range contractions or shifts as a result (Baltensperger and Huettmann 2015; Conradie et al. 2020). By measuring physiological and behavioural traits of thermoregulation in the heat (i.e., heat tolerance limits and evaporative cooling capacity) we can better understand how endotherms cope with high *T*_a_s and therefore improve conservation outcomes (Bronner et al. 1999; Reher et al. 2018; Noakes and McKechnie 2019).

The majority of research investigating thermal physiology of small endotherms in a climate change context has focussed on species inhabiting hot environments (Gerson et al. 2014; Smit and McKechnie 2015; Talbot et al. 2017; van Jaarsveld et al. 2021b). Predictably, endotherms from extremely hot environments exhibit higher heat tolerance limits and efficient evaporative cooling than species from temperate and mesic environments (Freeman et al. 2022). In birds under experimental conditions, Namaqua sandgrouse (*Pterocles namaqua*) can tolerate *T*_a_ = 60 ℃ (Czenze et al. 2021a), while red-billed queleas (*Quelea quelea*) can conserve water by tolerating body temperatures (*T*_b_) greater than 48 ℃ (Freeman et al. 2020). In mammals, Angolan free-tailed bats (*Mops condylurus*) can tolerate roost temperatures (*T*_roost_) of 60 ℃ (Bronner et al. 1999) and Australian free-tailed bats (*Ozimops* spp.) allow skin temperatures (*T*_skin_) to increase to 45.8 ℃ (Bondarenco et al. 2014). Despite these adaptations, many species are still overwhelmed by the increased frequency and intensity of extreme weather events such as heat waves (McKechnie and Wolf 2010; Bondarenco et al. 2014; Ratnayake et al. 2019; Pruvot et al. 2019).

Heat waves not only occur in arid, but also temperate regions (Stott et al. 2004; Xu et al. 2020; Brás et al. 2021; Schumacher et al. 2022; Sang and Hamann 2023) and their impact on small temperate endotherms may be disproportionately severe if these species have lower heat tolerance limits and evaporative cooling capacities. Recent studies on a cold specialised bird, the snow bunting (*Plectrophenax nivalis*), illustrate this species experiences heat stress at *T*_a_ below *T*_b_ and 95% of test subjects could not dissipate their entire metabolic heat production through evaporative cooling (O’Connor et al. 2021). Further, the mean heat tolerance limit of montane bird species was 2.5 ℃ lower than that of arid zone species (Freeman et al. 2022).

Research on the thermal physiology of temperate endotherms has focused on how animals survive lower *T*_a_s (Geiser and Brigham 2000; Jonasson and Willis 2011; Klüg-Baerwald and Brigham 2017; Geiser 2021; Chenery et al. 2022). In temperate areas, small endotherms like arctic ground squirrels (*Spermophilus parryii*) and hazel dormice (*Muscardinus avellanarius*) can allow their body temperatures to drop below 0 ℃, while temperate bats allow metabolic rates to drop as low as 2–5% of basal metabolic rates to save significant amounts of energy otherwise required to maintain normothermy (Barnes 1989; Geiser 2021; Pretzlaff et al. 2021). In Australia, vespertilionid bats use both short and long torpor bouts and can allow their *T*_b_ to drop as low as ~ 2 ℃ as *T*_a_ drops below freezing (Turbill and Geiser 2006, 2008; Stawski and Currie 2016; Currie et al. 2022). To further increase energy savings, temperate vespertilionid bats employ partial passive rewarming when rewarming from torpor (Turbill and Geiser 2006; Turbill 2008; Chenery et al. 2022). Compared to our understanding of how these animals tolerate and survive cold *T*_a_s, little is known about the heat tolerance of bats inhabiting colder climes and how they differ inter-sexually.

There is limited literature for endotherms that examine differences in the thermal physiology and heat tolerance limits of species based on their geographic distribution and sex. For example, desert populations of white-browed sparrow-weavers (*Plocepasser mahali*) exhibit higher heat tolerance limits and lower evaporative water loss (EWL) rates than mesic populations (Noakes et al. 2016). An arid population of house finches (*Carpodacus mexicanus*) maintained significantly lower EWL rates than their mesic counterparts (MacMillen and Hinds 1998). Additionally, differences in thermoregulatory capacities are also evident inter-sexually, with females of some vespertilionid species exhibiting higher heat tolerance limits than males. These females may be better adapted to conserve water until higher *T*_a_ (up to 42.5 ℃) potentially as a result of tolerating higher *T*_roosts_ and allocating water towards lactation during the breeding season (Czenze et al. 2022; de Mel et al. 2024).

When assessing geographical and inter-sexual variation in thermoregulation, the lesser long-eared bat (*Nyctophilus geoffroyi*) represents a good model species due to its cosmopolitan distribution and sexual differences in reproductive ecology such as the formation of maternity colonies by females while males roost alone. In this study, we evaluated *N. geoffroyi’s* heat tolerance from a cold area of their range during summer and tested the hypothesis that climate patterns influence species’ heat tolerance capacity during short-term exposure such as heat waves. We predicted that *N. geoffroyi* from relatively colder areas that are not water stressed, would not be under strong selection pressure to tolerate high *T*_a_s and would begin evaporative cooling at relatively low *T*_a_s, compared to desert conspecifics for which similar studies have been conducted. We also predicted that, similar to the patterns recorded by de Mel et al., (2024), females would have higher heat tolerance limits, and be more conservative with their water reserves than males due to their use of warmer summer maternity colonies and increased water requirements due to lactation.

## Methods

### *Study sites*

The study was conducted at Newholme field station (− 30. 415,362, 151. 636760) and Imbota nature reserve (− 30. 576,851, 151. 717,867) separated by 20 km, near Armidale NSW, Australia during the austral summers of 2022/2023, 2024 and 2024/2025. The sites range in elevation from 1000 to 1300 m. During the last 30 years for the period of December—March, the monthly mean maximum *T*_a_ ranged from 23.2 (March) to 26.3 (January) ℃, while the monthly mean minimum *T*_a_ ranged from 11.7 (March) to 13.6 (January) ℃ (BoM 2025). The highest *T*_a_ recorded for these months since 1993 has been 32.4, 36.5, 37.0 and 37.1 ℃ (20 March 2015, 02 December 2020, 03 January 2014 and 12 February 2017). The mean monthly rainfall for this period ranged from 69.7 to 102.1 mm. Water is available in the form of small streams and ponds distributed throughout the area. The dominant tree species at both sites are Eucalyptus and Acacia.

### *Bat capture and measurement of T*_skin_

Temperature telemetry was conducted during the Austral summer of 2022/2023. Bats were captured using mist nets (Ecotone, Gdynia, Poland) and harp traps (Faunatech Austbat, Victoria, Australia). Captured bats were aged, sexed and placed in clean cloth bags. We only used non-reproductive adults for experiments. Individuals were weighed to the nearest 0.1 g using an electronic weighing scale (Taylor digital scale, 1250 BK, China), and forearm length was measured to the nearest 0.1 mm.

We attached temperature sensitive radio-transmitters (~ 0.3 g, BD-2X, Holohil Systems Inc, Carp, ON, Canada) to five male (three from Newholme field station and two from Imbota nature reserve) and one female (from Imbota nature reserve) *N. geoffroyi* (7.8 ± 1.4 g), by clipping a small amount of fur between the scapulae and used a thin layer of adhesive [ADOS F2 contact adhesive, sensu (O’Donnell and Sedgeley 1999; Sedgeley and O’Donnell 1999)]. Prior to attachment, transmitters were calibrated to the nearest 0.1 ℃ between 5 and 50 ℃ in a water bath against a precision mercury thermometer (traceable to NIST). Skin temperature (*T*_skin_) of each individual was calculated from the transmitter pulse rate based on a polynomial regression calibration curve (R^2^ > 0.98). Ambient temperature was recorded using temperature loggers (Thermochron iButton, Maxim Integrated, San Jose, CA, USA, logging interval = 60 min) deployed in the shade at a height of 2 m (Stawski and Currie 2016).

After locating bats at their roosts each subsequent morning, a data logger [SRX-1200, SRX-800, Lotek, Newmarket, ON, Canada, and custom loggers sensu (Körtner and Geiser 1998)] was deployed near the roost. Transmitter pulse rates were obtained at 10 min intervals. Bats were considered to be torpid when *T*_skin_ was below 28 ℃ for more than 30 min (Bondarenco et al. 2016). This was based on a *T*_b_ of 31 ℃ as the torpor threshold and the difference between *T*_b_ and *T*_skin_ in small endotherms being < 3 ℃ (Barclay et al. 1996; Turbill et al. 2003). Active arousals from torpor were characterised by a sharp increase in *T*_skin_ above the torpor threshold while passive arousals were distinguished by *T*_skin_ closely following *T*_a_ (Fig. 2) during the initial part of arousal followed by active rewarming (Turbill et al. 2003; Bondarenco et al. 2016; Doty et al. 2016). A temperature data logger was deployed in accessible roosts (*n* = 2, 85 days per roost) to record *T*_roost_ after confirming it was unoccupied.

### *Gas exchange measurements*

Adult *N. geoffroyi* (10 males, 13 females) were transported to the University of New England in cloth bags and held in a temperature controlled dark room for < 24 h. Bats were offered water every morning and night and fed mealworms (*Tenebrio molitor*) until they gained ≥ 10% body mass or were satiated. A temperature sensitive PIT-tag (Biotherm, Biomark, Boise ID, USA, accuracy ± 0.5 ℃), which was calibrated to the nearest 0.1 ℃ between 5 and 50 ℃ in a water bath against a precision mercury thermometer (traceable to NIST), was implanted subcutaneously between the scapulae of each individual post feeding. Gas exchange measurements were commenced the morning after capture and six hours after feeding to ensure individuals were post-absorptive.

A flow-through respirometry system was used to measure evaporative water loss (EWL) and carbon dioxide (V̇_CO2_) during measurements. The airtight respirometry chambers, constructed from 0.5 L glass jars, included a wire mesh platform elevated ~ 2 cm from the bottom while the walls and top of the chamber were covered in mesh to allow bats to hang in natural roosting postures and crawl. A layer of mineral oil (~ 1 cm) on the bottom trapped faeces and urine excreted by the bat. The temperature within the respirometry chamber was measured using a copper-constantan thermocouple probe (0.1 ℃ accuracy) connected to a digital thermometer (Omega Handheld Digital Thermometer HH81A, Shanghai, China). The respirometry chamber was placed within a customised environmental chamber (WAECO mobile refrigeration unit: 14 L, TC-14FL, VIC, Australia) where temperature could be controlled manually. Subcutaneous temperature (*T*_sub_) readings from the PIT-tags were recorded continuously using a racket antenna adjacent to the respirometry chamber that was connected to an HPR plus reader (Biotherm, Biomark, Boise ID, USA).

Atmospheric air was passed through a column of silica using a low noise aquarium pump with a maximum capacity of 30 L min^−1^. The dried air was then split into baseline and chamber channels. We used a mass flow controller (MFC; model MC-10SLPM-D/5 m, Alicat Scientific Inc., Tucson AZ, USA) to regulate the chamber airflow and a needle valve to regulate baseline air flow. To maximise air mixing, the air inlet in the chamber was near the top of the chamber while the air outlet was below the mesh platform. Flow rates ranged from 0.6 to 2.6 L min^−1^ and were adjusted depending on behaviour at each *T*_a_. We used Bev-A-Line IV tubing (Thermoplastic Processes inc.) within the system. Excurrent air from the baseline and chamber was split using a multiplexor (MUX3-1101-18 M, Sable Systems, Las Vegas, NV, USA) in manual mode and then subsampled using a SS4 subsampler (Sable Systems) and was subsequently pulled through a CO_2_/H_2_O analyser (LI-850, LI-COR, Lincoln, NE, USA). The CO_2_/H_2_O analyser was calibrated for CO_2_ using a known CO_2_ concentration (2000 ppm) and was regularly zeroed using nitrogen. The H_2_O sensor of the LI-850 was also regularly spanned using air saturated with H_2_O at two dew point temperatures (see Marom et al. 2006) and zeroed using nitrogen. An analog–digital converter (UI-2, Sable Systems) was used to digitise the voltage outputs from the analysers and was recorded using Sable Systems Expedata software at a sampling interval of 5 s.

### *Experimental protocol*

Experiments, lasting 4.5–5.5 h in total, were conducted between 0700 and 1700 h (the diurnal rest phase of the bats) following Czenze et al., (2022). Animals were exposed to incrementally higher *T*_a_s in a stepped manner (Whitfield et al. 2015). This is analogous to the sliding cold exposure protocol used for summit metabolism measurements, where animals are exposed to a decreasing series of *T*_a_s, but in reverse (Swanson et al. 1996) and produces results indistinguishable from steady-state measurements without the harmful effects of prolonged exposure to high temperatures (Short et al. 2022). Individuals spent one hour at *T*_a_ = 28 ℃ to acclimate to the respirometry chamber before measurements. Recording was initiated at *T*_a_ = 28 ℃ and *T*_a_ was increased in 4 ℃ increments until 40 ℃ and then in 2 ℃ increments thereafter. At each *T*_a_, individuals were exposed to the experimental *T*_a_ for a minimum of 20–30 min and we waited until traces of *T*_sub_, V̇_CO2_, and EWL had stabilised at constant values before measuring. An infra-red video camera continuously monitored individuals for a suite of behaviours (i.e., prolonged ceaseless circular movements or escape behaviours, ceaseless chewing of the chamber), that indicated the individual had reached what we considered its ‘heat tolerance limit’ (Czenze et al. 2021b, 2022). We used this as the cut-off point to terminate the experiment and remove the individuals from the chamber. Bats were then weighed, offered water and placed in cloth bags to rest. Mealworms and more water were offered one hour later, and then again before being released at the site of capture.

### *Statistical analysis*

Analyser drift and lag were corrected using Expedata software. V̇_CO2_ and EWL were calculated using Eqs. 9.5 [V̇_CO2_ = Excurrent flow rate * (Fractional excurrent CO_2_) – Incurrent flow rate * (Fractional incurrent CO_2_)] and 9.6 [EWL = Excurrent flow rate * (Fractional excurrent H_2_O)—Incurrent flow rate * (Fractional incurrent H_2_O); Lighton 2008], from the lowest stable 5-min periods of CO_2_ and water vapour at each *T*_a_, assuming 0.803 mg H_2_O ml^−1^ vapour (Lighton 2008). A respiratory exchange ratio of 0.71 (Walsberg and Wolf 1995) was assumed to calculate resting metabolic rate (RMR) from V̇_CO2_ and V̇_CO2_ was converted to metabolic rate (W) assuming 27.8 J CO_2_ ml^−1^ (Withers 1992) since individuals were post-absorptive [We also analysed our data assuming an RER = 0.75 (80% fat, 20% protein) and RER = 0.83 (60% fat, 40% carbohydrate; Blaxter 1989; Schmidt-Nielsen 1997) resulting in no significant difference (RMSD = 0.005 and 0.012 respectively and R^2^ > 0.99), prompting us to use an RER of 0.71 to remain consistent with previous studies (Czenze et al. 2020b, 2022)]. Whole animal EWL (waEWL) and whole animal RMR (waRMR) was calculated for each individual and subsequently sex. Rates of waEWL were converted to evaporative heat loss (EHL, W) assuming a latent heat of vapourisation of water of 2.406 J mg^−1^ at 40 ℃ (Tracy et al. 2010). All statistical analyses were performed in R 4.4.2 (R Core Team, 2024). Inflection points of the physiological responses in relation to *T*_a_ were identified using the *SEGMENTED.LME* package (Muggeo 2016). We used an ANOVA to determine the best fit of the relationship (i.e., segmented or linear) for all physiological responses to *T*_a_. If the data best fit a segmented relationship, we considered a lack of overlapping confidence intervals to indicate a significant difference when comparing the inflection points between males and females. Linear mixed effect models were then fitted using the NLME package (Pinheiro et al. 2009) after the sex-specific inflection points. We included individual bat identity as a random factor to account for repeated measurements. *T*_a_, body mass (*M*_b_), sex and their interaction terms were used as predictor variables in the global models for each physiological variable. Based on null hypothesis testing, we selected the reduced predictor variables to be retained in the final model (Tredennick et al. 2021; Beilke and O’Keefe 2023). Estimated marginal means for *T*_sub_, waEWL, waRMR and EHL/metabolic heat production (MHP) were calculated using the emmeans package (Lenth 2021). Significance was assigned when *α* was < 0.05 or in cases where confidence intervals did not overlap.

## Results

### *T*_skin_*measurements*

The mass of the males used in the temperature telemetry experiments was 7.6 ± 1.5 g while the mass of the female was 8.8 g. We collected 49 bat-days of temperature telemetry data (3–11 days per individual). On two days bats roosted in tree hollows, but were found roosting under exfoliating bark on all other days. On one day we found a bat roosting in the hollow of a fallen tree trunk ~ 50 cm from the ground. The mean ± SD daily maximum *T*_skin_ = 35.2 ± 4.5 ℃, absolute maximum *T*_skin_ = 40.9 ℃, and absolute minimum *T*_skin_ = 8.4 ℃ (Table 1). The mean ± SD daily maximum *T*_a_ = 25.0 ± 3.5 ℃ (absolute maximum = 33.5 ℃, absolute minimum = 5 ℃) while the mean daily maximum *T*_roost_ = 28.3 ℃ (absolute maximum = 35.4 ℃, absolute minimum = 6.6 ℃).

**Table 1:** Summary of *T*_skin_ for adult *N. geoffroyi* (five males, one female), *T*_roost_ (*n* = 2) and *T*_a_ during November 2022—March 2023 at Newholme field laboratory and Imbota nature reserve, Armidale, NSW, Australia. All values are in ℃. Mean ± SD are reported

	*T*_skin_	*T*_roost_	*T*_a_
Mean daily minimum	19.1 ± 6.5	16.6	12.3 ± 2.8
Absolute minimum	8.4	6.6	5
Mean daily maximum	35.2 ± 4.5	28.3	25.0 ± 3.5
Absolute maximum	40.9	35.4	33.4
Maximum daily range	16.0 ± 6.2	11.6	12.7 ± 3.6

**Fig. 1:**
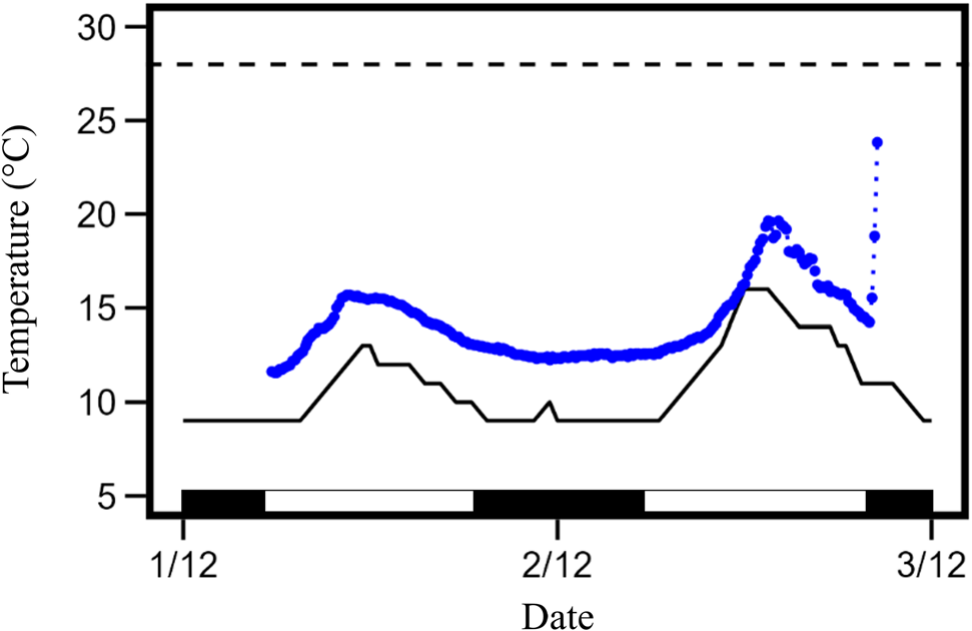
Skin temperature (blue circles) during a torpor bout of a male *Nyctophilus geoffroyi* at Newholme, NSW, Australia during December 2022. Solid black line represents shaded air temperature while horizontal dashed line represents torpor threshold. Black and white bars at the bottom denote night and day respectively

**Fig. 2:**
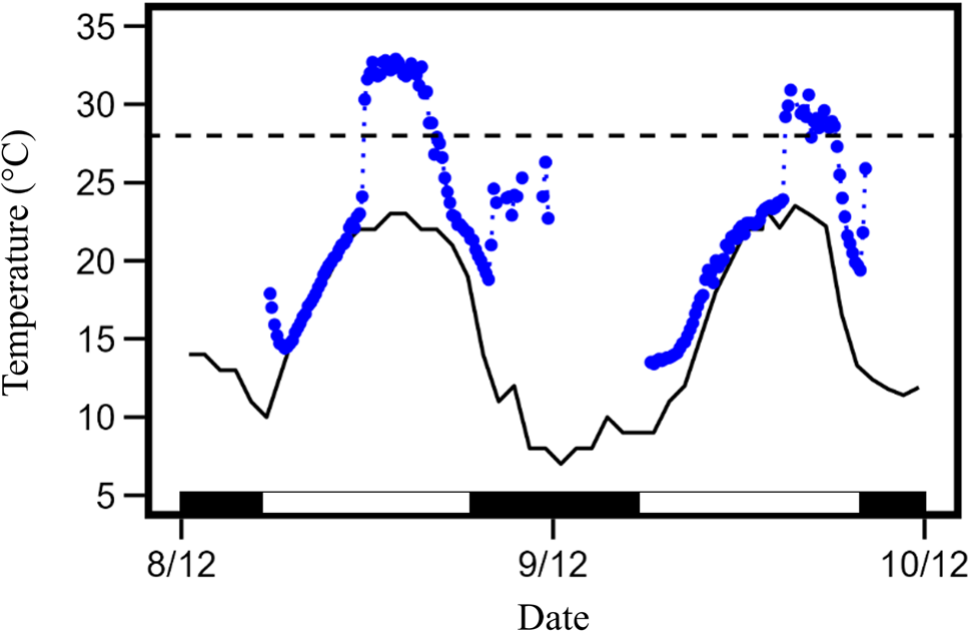
Skin temperature (blue circles) of a male *Nyctophilus geoffroyi* indicating torpor bouts on two consecutive days from Newholme, NSW, Australia during December 2022. Unbroken black line represents shaded air temperature while horizontal dashed line represents torpor threshold. Black and white bars at the bottom denote night and day respectively

We recorded at least one torpor bout on all but one bat day (21 January 2023, *T*_a_ range = 11.5–24.8 ℃) totalling 61 torpor bouts with 49 beginning immediately before dawn. We recorded 40 arousals during daytime of which 11 were active while 29 included some degree of passive rewarming. The mean ± SD *T*_a_ at torpor entry was 14.2 ± 4.6 ℃. The mean ± SD torpor duration was 307 ± 323 min with the longest torpor bout = 28.83 h (Fig. 1). On 19 bat days, only one torpor bout was recorded while on 14, two were recorded (Fig. 2). Three torpor bouts occurred on three bat days.

### *Respirometry*

**Fig. 3:**
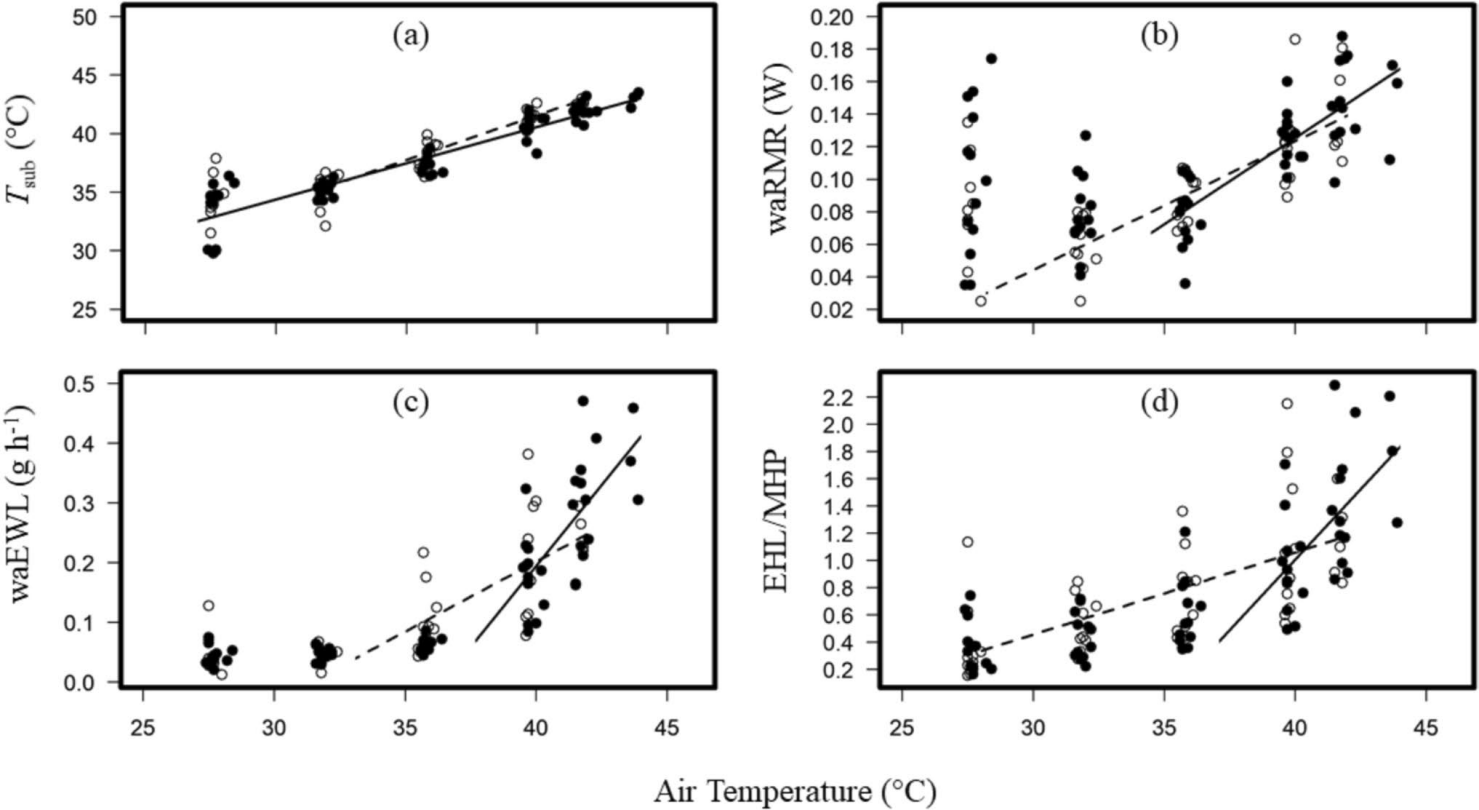
(**a**) Subcutaneous body temperature (*T*_sub_), (**b**) whole animal resting metabolic rate (waRMR), (**c**) whole animal evaporative water loss (waEWL) and (**d**) evaporative heat loss (EHL)/metabolic heat production (MHP) at high air temperatures of male (*n* = 10) and female (*n* = 13) Lesser long-eared bats (*Nyctophilus geoffroyi*). Dashed and solid lines represent regressions for males and females respectively above inflection points (Table 2). Open circles indicate males and filled circles indicate females

The mean ± SD of the mass of females was 8.9 ± 1.0 g while for males it was 7.3 ± 0.9 g. However, Mass was not a significant predictor variable for any of the models tested. The final reduced mixed effect models for *T*_sub_ retained *T*_a_ and *Sex* (Table 3). Female *N. geofforyi* could tolerate *T*_a_ = 44 ℃ (*n* = 3) while males could only tolerate *T*_a_ = 42 ℃ (*n* = 5). Females and males tolerated similar maximum *T*_sub_ (ANOVA: f = 0.025, *p* = 0.87; females = 42.9 ± 0.7 ℃, males = 42.6 ± 0.3 ℃). The *T*_sub_ of females increased linearly with *T*_a_ displaying no inflection point while there was an inflection point for males (32.1 ℃, CI = 30.2–33.9 ℃; Fig. 3a).

The final reduced mixed effect models for waRMR retained the interaction term *T*_a_ * *Sex* (Table 3). Both sexes displayed an inflection point for waRMR, which was significantly higher for females (*n* = 13; 34.5 ℃, 33.1–35.9 ℃) than males (*n* = 10; 28.2 ℃, 27.6–28.8 ℃; Table 2). Above the inflection point, females increased waRMR more quickly (10 mW ℃^−1^) than males (7 mW ℃^−1^; Fig. 3b), although the maximum waRMR achieved by both sexes were similar (females = 0.15 W, males = 0.14 W).

**Table 2:** Summary of thermoregulatory performance in *Nyctophilus geoffroyi* captured at Imbota nature reserve, Armidale, NSW, Australia. Thermoregulatory variables include subcutaneous temperature (*T*_sub_), whole animal resting metabolic rate (waRMR), whole animal evaporative water loss (waEWL) and evaporative heat loss/metabolic heat production (EHL/MHP). ‘Max’ and ‘Min’ indicate maximum and minimum values for each variable respectively. Mean values are reported ± SD with sample sizes in parentheses. Inflection points reported with 95% confidence intervals

	Female	Male
Mass (g)	8.9 ± 1.0 (13)	7.3 ± 0.9 (10)
Subcutaneous temperature (*T*_sub_)		
Min *T*_sub_ (℃)	33.4 ± 2.5 (13)	34.6 ± 1.8 (10)
Inflection *T*_a_ (℃)	NA	32.1 (30.2 – 33.9)
Max *T*_sub_ (℃)	42.9 ± 0.7 (3)	42.6 ± 0.3 (5)
* T*_sub_ slope (℃ ℃^−1^)	0.61	0.75
Max experimental* T*_a_ (℃)	44 (3)	42 (5)
Whole animal resting metabolic rate (waRMR)		
Min waRMR (W)	0.08 ± 0.02 (13)	0.06 ± 0.02 (10)
Inflection* T*_a_ (℃)	34.5 (33.1—35.9)	28.2 (27.6 – 28.8)
Max waRMR (W)	0.15 ± 0.03 (3)	0.14 ± 0.03 (5)
waRMR slope post inflection point (mW ℃^−1^)	10	7
Max waRMR/min waRMR	1.88	2.33
Whole animal evaporative water loss (waEWL)		
Min waEWL (g/h)	0.05 ± 0.02 (13)	0.04 ± 0.03 (10)
Inflection *T*_a_ (℃)	37.7 (36.9 – 38.6)	33.1 (31.3 – 34.8)
Max waEWL (g/h)	0.38 ± 0.08 (3)	0.23 ± 0.05 (5)
waEWL slope post inflection point (mg h^−1^ ℃^−1^)	54	23
Max waEWL/min waEWL	7.6	5.8
Max EHL/MHP	1.76 ± 0.47 (3)	1.15 ± 0.31 (5)

The final reduced mixed effect models for waEWL retained the interaction term *T*_a_ * *Sex* (Table 3). Both sexes displayed an inflection point for waEWL, with the female inflection point (37.7 ℃, 36.9–38.6 ℃) significantly higher than that of males (33.1 ℃, 31.3–34.8 ℃; Table 2). Above the inflection point, females increased waEWL (54 mg h^−1^ ℃^−1^) twice as quickly as males (23 mg h^−1^ ℃^−1^; Table 2, Fig. 3c). The maximum EHL/MHP of females (1.76 ± 0.47) was higher than males (1.15 ± 0.31; Table 2) meaning they could dissipate 176% of the heat they produced metabolically compared to only 115% for males (Fig. 3d).

**Table 3:** Final reduced linear mixed effect model output of thermoregulatory performance as a function of chamber air temperature (*T*_a_) in lesser long eared bats (*Nyctophilus geoffroyi*) from Imbota nature reserve, NSW, Australia. Thermoregulatory variables include subcutaneous temperature (*T*_sub_), whole animal resting metabolic rate (waRMR), whole animal evaporative water loss (waEWL) and evaporative heat loss/metabolic heat production (EHL/MHP). Numbers in parenthesis = (no. of observations, no. of individuals). Interactions between fixed effects denoted with ‘*x*’

Dependant variable	R^2^	Fixed effect	df	*t*	*p*
*T*_sub_ (92, 23)	0.88	*T*_a_	73.08	24.77	< 0.0001
		Sex (Male)	30.65	2.29	0.02
waRMR (75, 23)	0.77	*T*_a_	53.68	10.06	< 0.0001
		Sex (Male)	54.53	2.20	0.031
		*T*_a_ *x* Sex	53.31	-2.172	0.034
waEWL (52, 23)	0.77	*T*_a_	29.51	7.514	< 0.0001
		Sex (Male)	29.65	3.397	0.001
		*T*_a_ *x* Sex	29.23	-3.409	0.001
EHL/MHP (72, 23)	0.77	*T*_a_	51.75	4.68	< 0.0001
		Sex (Male)	52.18	2.955	0.004
		*T*_a_ *x* Sex	51.61	-2.978	0.004

## Discussion

We predicted that cold-adapted small endotherms would possess limited heat tolerance and rely heavily on evaporative cooling compared to their arid counterparts. Our results support these predictions with the study population of *N. geoffroyi* possessing lower heat tolerance limits and both males and females using evaporative cooling at much lower *T*_a_s than arid and semi-arid populations. Also, as predicted and consistent with conspecifics from arid and semi-arid ecosystems, we found that females had a higher heat tolerance limit and were more conservative with their water reserves than males. Overall, our results support the contention that reproductive pressures and climatic conditions are two major factors influencing the thermal physiology of small endotherms.

The prevalent use of torpor in summer has been reported for *N. geoffroyi* and other temperate bat species (Audet and Fenton 1988; Chruszcz and Barclay 2002; Turbill et al. 2003) allowing them to save significant amounts of energy and water (Cryan and Wolf 2003; Bondarenco et al. 2016). Torpor bout duration is often driven by ambient temperatures (Geiser 2021). Given the colder *T*_a_s experienced by this population, the duration of torpor bouts were predictably longer than in a desert population of *N. geoffroyi* (99 ± 52 min; de Mel et al. 2024). However, the longest torpor bout we recorded was nearly 30% shorter than the longest bout recorded by Turbill et al., (2003), illustrating the large variation in the expression of torpor in this population.

The mean daily maximum *T*_roost_ (27.7 ± 4.8 ℃) we recorded were relatively mild with only three instances of *T*_a_ above the torpor threshold *T*_b_ (31 ℃). Such low *T*_a_s do not allow bats to rewarm entirely passively like their desert counterparts (Bondarenco et al. 2013; de Mel et al. 2024), but necessitates active rewarming. Active arousals from torpor are 50% more energetically costly than passive arousals (Currie et al. 2015). More than 70% of our observed day time arousals were passive to some extent (Fig. 2), indicating a tendency for these individuals to minimise energy expenditure during rewarming as much as possible much like other species such as elephant shrews (*Elephantulus myurus*; Mzilikazi et al. 2002), stripe-faced dunnarts (*Sminthopsis macroura*; Geiser and Drury 2003) and Australian owlet-nightjar (*Aegotheles cristatusrs*; Doucette et al. 2011). Since torpid metabolic rate is positively correlated with torpid *T*_a_ (Geiser 2021), if daily temperatures increase due to climate change, bats like *N. geoffroyi* that prefer poorly insulated roosts (e.g., under bark) will expend more energy during their rest phase. In future climate scenarios, if individuals choose roosts that are exposed to direct solar radiation they may be able to offset some increases in torpid energy expenditure by reducing arousal costs via passive rewarming to higher temperatures. However, we recorded a maximum *T*_roost_ of 35.4 ℃ (corresponding *T*_a_ = 29.6 ℃; Table 1), due to direct solar exposure and in the future, higher *T*_roost_s might expose bats to acute hyperthermia, which underscores the delicate balance of thermoregulation in the heat even in colder ecosystems.

The *T*_b_ inflection for males (32.1 ℃, CI = 30.2–33.9 ℃) was similar to males from arid (33.2 ℃, CI = 32.5–36.1 ℃) and semi-arid (33.3 ℃, CI = 32.8–34.3 ℃) ecosystems (de Mel et al. 2024; de Mel et al., unpublished data) while females did not display an inflection. Given the low *T*_a_s of this region (Table 1; BoM, 2025) an undefended increase in *T*_sub_ with *T*_a_ would help conserve energy as individuals are not defending a set point temperature across a wide range of temperatures (Bondarenco et al. 2013; Currie et al. 2015). Thus, together with our temperature telemetry data, our respirometry data suggest that *N. geoffroyi* of this region, are adapted to maximise energy savings when rewarming during low *T*_a_s.

All females from our study could tolerate a *T*_a_ of 42 ℃, but only 50% (5/10) of males could tolerate a *T*_a_ of 42 ℃ (Table 2), demonstrating a higher heat tolerance limit in females. Such intersexual discrepancy in heat tolerance limits has been previously reported from southern yellow-billed hornbills (*Tockus leucomelas*; van Jaarsveld et al. 2021a), *Nyctalus noctula* from Europe (Czenze et al. 2022) and other populations of *N. geoffroyi* (de Mel et al. 2024; de Mel et al. unpublished data). In *T. leucomelas*, this is due to the inability of the females to behaviourally thermoregulate while entombed in tree hollows during incubation and rearing of chicks (van Jaarsveld et al. 2021a). In *Nyctalus noctula* and *N. geoffroyi*, the authors ascribed these discrepancies to sex differences in reproductive ecology (Czenze et al. 2022; de Mel et al. 2024) as females, like many other vespertilliods, form maternity colonies while males roost solitarily or in small groups (Turbill and Geiser 2006; Churchill 2008; Turbill et al. 2020). Maternity roosts are often warmer than solitary roosts (Kerth et al. 2001; Rensel et al. 2022), which is exacerbated by the endogenous heat production of the colony (Willis and Brigham 2007). While females contend with these conditions for much of summer, males roost solitarily (Turbill et al. 2003; Churchill 2008) and experience lower temperatures.

Females began using more energy at 34.5 ℃ (CI: 33.1–35.9 ℃), which was significantly higher than the corresponding value for males (28.2 ℃, CI = 27.6–28.8 ℃; Table 2). However, these inflection points are considerably lower than either sex from a desert population (females = 39.6 ℃, males = 38.5 ℃; de Mel et al. 2024). This suggests that this population of *N. geoffroyi*, when thermoregulating, begin to actively respond to heat when *T*_a_ is below normothermic *T*_b_; a phenomenon observed in other small endotherms adapted to cooler environments (Cortés et al. 2000; O’Connor et al. 2021). Increased energy expenditure during rest must be offset by increased feeding during the active phase or individuals may experience sub-lethal fitness costs like progressive *M*_b_ loss over successive warm days (du Plessis et al. 2012; van de Ven et al. 2019; Kemp et al. 2020). Given the incremental trajectory of global warming (Dosio et al. 2018; IPCC 2021), such threats could become pronounced in the future particularly as increased metabolic demands are associated with concomitant water costs (Conradie et al. 2019).

A significantly higher waEWL inflection point in females compared to males as was evident in our study (females: 37.7 ℃, CI = 36.9–38.6 ℃; males: 33.1 ℃, CI = 31.3–34.8 ℃) has also been reported in *Nyctalus noctula*, *Pipistrellus pygmaeus* and *P. pipistrellus* from Europe (Czenze et al. 2022). A higher waEWL inflection point allows females to conserve water until it is imperative, and thereby allocate water for lactation (Czenze et al. 2022; de Mel et al. 2024), which places a high demand on water reserves (Kurta et al. 1989, 1990; Adams and Hayes 2008). Despite delaying evaporative cooling by more than 4 ℃ than males, females still managed to dissipate 100% of MHP within 1 ℃ of males, further demonstrating their capacity to rapidly cool themselves using conserved water (Fig. 3d, Table 3, Table S1). Conversely, males using evaporative cooling at lower *T*_a_s could be reflective of evolutionary pressures towards increasing reproductive fitness as exposure to chronic heat stress results in increased apoptosis of sperm cells and decrease in testicular mass and volume in small endotherms (Setchell 2006; Wechalekar et al. 2016; Jacobs et al. 2024). Interestingly, this difference between male and female *N. geoffroyi* we measured here is consistent in both arid and semi-arid ecosystems, further emphasising the presence of similar reproductive pressures across populations (de Mel et al. 2024; de Mel et al., unpublished data). We recommend future studies to, when possible, take measurements from both sexes to explore how sexual selection pressures impact thermoregulation in the heat.

The heat tolerance and evaporative cooling capacity of individuals recorded in this study varied to those collected from other populations using identical methodology. For example, the heat tolerance limits of this population (females = 44 ℃, males = 42 ℃) were less than 4 ℃ than the respective sexes from an arid population of *N. geoffroyi* (females = 48 ℃, males = 46 ℃; de Mel et al. 2024), underscoring how small endotherms inhabiting comparatively cooler ecosystems and microhabitats are less tolerant of high temperatures (Czenze et al. 2021b; Freeman et al. 2022). Arid zone populations are under greater pressure to conserve water and this is exemplified by the minimum water loss rates recorded from the arid population of *N. geoffroyi* being 200–250% lower than the population studied here and 270–300% less at *T*_a_ of 40 ℃ (de Mel et al. 2024). This, combined with the temperature at which males from this study begin to evaporatively cool themselves (33.1 ℃) being nearly 10 ℃ lower than that of females from the arid zone (42.5 ℃; de Mel et al. 2024), underpins the argument that inter-population differences in thermal physiology can be driven by climatic conditions (MacMillen and Hinds 1998; Reher et al. 2022). The abundant water sources in the current study site likely allows this population to predictably replenish any water lost via daily evaporative cooling and may facilitate the maintenance of such high EWL rates that would be untenable in more arid ecosystems (Czenze et al. 2020a). We recommend research focussing on the drivers of these inter-population differences to determine the degree to which they can be attributed to inherent molecular-genetic differences and/or phenotypic plasticity.

## Conclusion

We provide compelling support to the notion that climatic differences among populations and reproductive pressures within populations appear to influence the thermoregulatory capacities of small endotherms. Although temperate populations, compared to arid and semi-arid populations are relatively buffered from extreme weather events like heatwaves, future climate change could still lead to sub-lethal fitness costs in these cold adapted endotherms.

## Supplementary Information

Below is the link to the electronic supplementary material.

Supplementary file1 (DOCX 16 KB)

## Data Availability

Data available from corresponding author upon reasonable request.
