# Supplementary material for: How to keep your cool: heat tolerance and thermoregulatory strategies of a cold adapted insectivorous bat
Source: Oecologia. 2025 Jul 28;207(8):136. doi: 10.1007/s00442-025-05776-3 (PMC12304034; doi:10.1007/s00442-025-05776-3)
Supplement: Supplementary file 1 — Supplementary file1 (DOCX 16 KB) [file 442_2025_5776_MOESM1_ESM.docx]

Title: How to keep your cool: Heat tolerance and thermoregulatory strategies of a cold adapted insectivorous bat

Table S1. Comparison of subcutaneous body temperature (*T*_sub_), whole animal resting metabolic rate (waRMR), whole animal evaporative water loss (waEWL) and evaporative cooling efficiency (EHL/MHP) at high air temperatures (*T*_a)_ between female (F) and male (M) *Nyctophilus geoffroyi*. Estimated marginal means (EMM) and SE values presented.

|  | Sex | EMM | SE | df | Lower CL | Upper CL |
| --- | --- | --- | --- | --- | --- | --- |
| *T*_sub_ |  |  |  |  |  |  |
| 28 | F | 33.2 | 0.269 | 54.0 | 32.6 | 33.7 |
|  | M | 33.9 | 0.286 | 43.0 | 33.3 | 34.5 |
| 32 | F | 35.6 | 0.220 | 26.7 | 35.2 | 36.1 |
|  | M | 36.3 | 0.248 | 25.7 | 35.8 | 36.9 |
| 36 | F | 38.1 | 0.204 | 19.7 | 37.7 | 38.5 |
|  | M | 38.8 | 0.243 | 23.4 | 38.3 | 39.3 |
| 40 | F | 40.5 | 0.229 | 30.4 | 40.1 | 41.0 |
|  | M | 41.2 | 0.272 | 35.4 | 40.7 | 41.8 |
| 42 | F | 41.8 | 0.254 | 43.3 | 41.2 | 42.3 |
|  | M | 42.5 | 0.297 | 47.2 | 41.9 | 43.1 |
| waRMR |  |  |  |  |  |  |
| 28 | F | -0.00467 | 0.01297 | 62.6 | -0.0306 | 0.0213 |
|  | M | 0.02862 | 0.00874 | 70.8 | 0.0112 | 0.0461 |
| 32 | F | 0.03893 | 0.00900 | 70.0 | 0.0210 | 0.0569 |
|  | M | 0.06027 | 0.00619 | 46.3 | 0.0478 | 0.0727 |
| 36 | F | 0.08254 | 0.00561 | 50.1 | 0.0713 | 0.0938 |
|  | M | 0.09193 | 0.00485 | 20.0 | 0.0818 | 0.1020 |
| 40 | F | 0.12615 | 0.00444 | 22.9 | 0.1170 | 0.1353 |
|  | M | 0.12358 | 0.00565 | 34.0 | 0.1121 | 0.1351 |
| 42 | F | 0.14796 | 0.00529 | 40.3 | 0.1373 | 0.1586 |
|  | M | 0.13941 | 0.00669 | 53.2 | 0.1260 | 0.1528 |
| waEWL |  |  |  |  |  |  |
| 28 | F | -0.40443 | 0.0883 | 31.4 | -0.5844 | -0.22447 |
|  | M | -0.08194 | 0.0454 | 41.5 | -0.1737 | 0.00978 |
| 32 | F | -0.20476 | 0.0625 | 34.2 | -0.3317 | -0.07777 |
|  | M | 0.01323 | 0.0328 | 48.0 | -0.0527 | 0.07917 |
| 36 | F | -0.00508 | 0.0380 | 43.4 | -0.0817 | 0.07156 |
|  | M | 0.10841 | 0.0236 | 27.5 | 0.0601 | 0.15671 |
| 40 | F | 0.19459 | 0.0203 | 25.8 | 0.1529 | 0.23634 |
|  | M | 0.20359 | 0.0224 | 22.9 | 0.1571 | 0.25004 |
| 42 | F | 0.29443 | 0.0208 | 26.9 | 0.2518 | 0.33704 |
|  | M | 0.25118 | 0.0256 | 34.7 | 0.1992 | 0.30312 |
| EHL/MHP |  |  |  |  |  |  |
| 28 | F | -1.082 | 0.4913 | 53.9 | -2.067 | -0.097 |
|  | M | 0.333 | 0.1102 | 27.2 | 0.107 | 0.559 |
| 32 | F | -0.386 | 0.3458 | 56.8 | -1.079 | 0.306 |
|  | M | 0.577 | 0.1004 | 18.9 | 0.367 | 0.788 |
| 36 | F | 0.310 | 0.2060 | 65.5 | -0.101 | 0.721 |
|  | M | 0.822 | 0.0991 | 17.9 | 0.613 | 1.030 |
| 40 | F | 1.006 | 0.1000 | 30.6 | 0.802 | 1.210 |
|  | M | 1.066 | 0.1067 | 24.0 | 0.846 | 1.286 |
| 42 | F | 1.354 | 0.1026 | 31.5 | 1.145 | 1.563 |
|  | M | 1.188 | 0.1134 | 30.0 | 0.957 | 1.420 |
|  |  |  |  |  |  |  |
